# Identification of Immune Response to Sacbrood Virus Infection in *Apis cerana* Under Natural Condition

**DOI:** 10.3389/fgene.2020.587509

**Published:** 2020-10-26

**Authors:** Yanchun Deng, Hongxia Zhao, Shuo Shen, Sa Yang, Dahe Yang, Shuai Deng, Chunsheng Hou

**Affiliations:** ^1^Institute of Apicultural Research, Chinese Academy of Agricultural Sciences, Beijing, China; ^2^Key Laboratory of Pollinating Insect Biology, Ministry of Agricultural and Rural Affairs, Beijing, China; ^3^Graduate School of Chinese Academy of Agricultural Sciences, Beijing, China; ^4^Guangdong Key Laboratory of Animal Conservation and Resource Utilization, Guangdong Public Laboratory of Wild Animal Conservation and Utilization, Guangdong Institute of Applied Biological Resources, Guangdong Academy of Science, Guangzhou, China; ^5^Qinghai Academy of Agriculture and Forestry Sciences (Academy of Agriculture and Forestry Sciences), Qinghai University, Xining, China

**Keywords:** Chinese sacbrood virus, serine protease, *Apis cerana*, transcriptome, siRNA, immune response

## Abstract

Chinese sacbrood virus (CSBV) is a serious threat to eastern honeybees (*Apis cerana*), especially larvae. However, the pathological mechanism of this deadly disease remains unclear. Here, we employed mRNA and small RNA (sRNA) transcriptome approach to investigate the microRNAs (miRNAs) and small interfering RNAs (siRNAs) expression changes of *A. cerana* larvae infected with CSBV under natural condition. We found that serine proteases involved in immune response were down-regulated, while the expression of siRNAs targeted to serine proteases were up-regulated. In addition, CSBV infection also affected the expression of larvae cuticle proteins such as larval cuticle proteins A1A and A3A, resulting in increased susceptibility to CSBV infection. Together, our results provide insights into sRNAs that they are likely to be involved in regulating honeybee immune response.

## Introduction

The Asian honeybee, *Apis cerana*, is an important pollinator to maintain plant biodiversity in Southeast Asian countries, especially to wild flowering plants and crops (Ai et al., 2012). However, honeybees have been suffering colony decline in recent years due to an increasing number of infections from multiple pathogens (Diao et al., 2018a). Among honeybee pathogens, Chinese sacbrood virus (CSBV), a Chinese strain of sacbrood virus (SBV), is the major factor threatening the survival of *A. cerana* colony (Sun et al., 2018). SBV is the single-strand positive RNA virus, which can infect honeybee larvae and lead to larvae death (Chen and Siede, 2007). SBV belongs to the genus *Iflavirus*, the family Iflaviridae within the order Picornavirales (Bailey et al., 1964). The full-length genome of SBV was sequenced in 1999 (Ghosh et al., 1999).

The clinical symptom of CSBV-infected *A. cerana* larvae was ecdysial fluid aggregated with typical “sac,” resulting in failure to pupate (Ma et al., 2013). CSBV was identified firstly from *A. cerana* larvae in Guangdong province of China in 1972 (Yang et al., 1988), and frequently caused extensive larvae death and colony decline and recently re-emerged as one of the causative agents of larvae disease outbreaks in Liaoning province in 2008 (Ma et al., 2011). Since then, CSBV is frequently detected in *A. cerana* and remains a major threat to *A. cerana* in China (Hu et al., 2016; Diao et al., 2018a). Usually, viral infection can induce host cell apoptosis and tissue damage as well as functional disorder of target genes. Then, all these alterations can be displayed through gene expression changes (Tan et al., 2007; Francis et al., 2012).

Like many other social insects, honeybees have no adaptive immune response known in vertebrates (Evans and Armstrong, 2006). To survive under the persistent viral infection, honeybees have evolved several defense mechanisms that are activated via different immune pathways (Evans et al., 2006; Zou et al., 2006). Honeybee antiviral defense mechanisms include RNA interference (RNAi), Janus kinase/Signal Transducer and Activator of Transcription (JAK/STAT) pathway, Toll pathway, Immune Deficiency (Imd) pathway, c-Jun N-terminal kinase (JNK) pathway, Mitogen-Activated Protein Kinases (MAPK) pathway and melanization, as well as reactive oxygen species generation (Brutscher et al., 2015). Serine protease (SP) and serine protease homolog (SPH) play an important role in innate immune response that includes coagulation, melanization, and the Toll signaling pathway in honeybee (Zou et al., 2006; Gao et al., 2017).

Experimental evidence has shown that CSBV infection induced routine immune responses by enhancing the expression of antimicrobial peptides (AMPs) (Liu et al., 2017). Generally, activation of the Toll, Imd and JAK/STAT pathways result in the production of AMPs and other effector proteins (Brutscher et al., 2015). Nevertheless, knowledge about the comprehensive immune responses to CSBV infection is limited. Omics technique is a useful tool to measure the dynamic changes of whole gene expression associated to viral infection (Sheyn et al., 2018). Using the proteomic technique, it was found 142 proteins and 12 phosphoproteins down-regulated in CSBV-infected larvae, which were significantly affected in carbohydrate, energy and fatty acid metabolism (Han et al., 2013). Lately, the full-genome sequence of *A. cerana* was determined, providing new insights into physiological resistance to Varroa mites, and was found to have six more immune genes than *Apis mellifera* (Diao et al., 2018b). In addition, transcriptome technology was applied to several fields of honeybees, such as gland development (Liu et al., 2014), Varroa mite control (Campbell et al., 2015), and *Ascosphaera apis* pathogenesis (Cornman et al., 2012). Previous studies have confirmed that chemical stressors and pathogens can lead to up-regulate of AMPs (Siede et al., 2012; Li et al., 2016). Galbraith et al. (2015) identified the transcript and epigenetic responses of honeybees to Israeli acute paralysis virus (IAPV) infection and found that honeybees employed distinct immune pathways such as JAK-STAT to resistance viral infection besides universal immune responses. Recently, Rutter et al. (2019) confirmed that IAPV infection combined with diet quality can affect the immune gene expression of Notch and JAK-STAT signaling pathways. Nevertheless, artificial infection with acute bee paralysis virus (ABPV) did not induce a humoral immune response of larvae and workers (Azzami et al., 2012). In addition, artificial infection frequently caused the unexpected transcription changes of AMPs as well as non-target genes (Boncristiani et al., 2013).

Small RNAs (sRNAs) include microRNAs (miRNAs) and small interfering RNAs (siRNAs) that are involved in regulating gene expression in most organisms (Ding and Voinnet, 2007; Flenniken and Andino, 2013; Fletcher et al., 2016). miRNAs are a group of sRNAs with 22 nt in size and are important regulators of diverse biological processes, including development and interactions between host and virus (Ding and Voinnet, 2007). siRNA is produced by double-stranded RNA (dsRNA), which can be from either the viral genome itself or an intermediate dsRNA product generated during viral replication (Fung et al., 2018). Deep sequencing on honeybee samples found that vsiRNAs were matched to several honeybee viruses such as Deformed wing virus (DWV) and IAPV (Chejanovsky et al., 2014; Fung et al., 2018). Virus-derived small interfering RNAs (vsiRNA) produced during the viral infection are a group of siRNA ranged from 21 to 24 nt in size that may hijack the host RNAi pathway (Aliyari et al., 2008). In brief, vsiRNA guides the RNA induced silencing complex (RISC) to target viral genomes, which can potentially alter the host transcriptome responses (Ding, 2010; Yang et al., 2018). However, little research has been done on the sRNAs from CSBV. Thus, the mechanisms underlying the host responses to CSBV infection are unknown, especially the effects of sRNAs on CSBV infection under natural condition. Here, we examined the transcriptional responses and the abundance of siRNAs of *A. cerana* larvae to CSBV infection under natural conditions. We characterized (1) the transcriptomic variation of genes related to larval development and immune, (2) the host’s metabolism, and (3) determined whether there is a relationship between siRNA and CSBV infection. Our results provide insights into up- and down-regulated in cuticle protein and serine proteinase during CSBV infection. These findings significantly broaden our knowledge of virus-host interactions and provide novel targets for the control of CSBV.

## Materials and Methods

### Sample Collection

The samples were collected from Liaoning and Guangdong provinces in October 2016. The larvae of *A. cerana* were collected from three apiaries in different regions (Longmen, Meizhou, and Conghua) in Guangdong province in China. CSBV-infected larvae were taken from the colonies with obvious cystic symptoms and confirmed it using RT-PCR with a pair of specific primers, 5′-CCTGGGAAGTTTGCTAGTATTTACG-3′ and 5′-CCTATCACATCCATCTGGGTCAG-3′ according to the described by Ma et al. (2013) and the healthy larvae were collected from three different apiaries (Huludao and Benxi in Liaoning Province, and Guangzhou in Guangdong province). For RNA sequencing, the larvae of *A. cerana* were mixed into three groups with the same genetic background. To avoid interferon by other viruses, six common honeybee viruses were tested before RNA sequencing (Supplementary Table 1).

The 2 or 3-day-old larvae were used for transcriptome analysis since they were most susceptible to CSBV (Sun et al., 2018). The age of larvae was identified by confining a queen to lay eggs within 24 h. Twenty 2-day-old larvae were considered as one treatment group, and each group consisted of three replicates. However, only two group samples were used for further analysis due to the RNA-seq data quality. These samples alive were taken and then immediately transferred into liquid nitrogen until use. In addition, naturally CSBV-infected and healthy larvae (4-, 5-, 6-, and 7-day-old) were used to identify the expression of the vsiRNA and immune genes.

### RNA Extraction, Library Construction, and RNA-seq

Total RNA of each sample were isolated from the larvae using the Trizol Reagent (Life technologies, California, CA, United States). RNA quality was measured using an Agilent 2100 Bioanalyzer (Agilent Technologies, Inc., Santa Clara, CA, United States). The mRNA was obtained by NEBNext Poly (A) mRNA Magnetic Isolation Module (NEB, Ipswich, MA, United States). Briefly, the enriched mRNA was fragmented into approximately 200-nt RNA inserts, which were used to synthesize the cDNA. The end-repair/dA-tail and adaptor ligation were performed to the double-stranded cDNA. The corresponding fragments were obtained by Agencourt AMPure XP beads (Beckman Coulter, Inc., United States) and PCR amplification. PCR was performed by using Phusion High-Fidelity DNA polymerase, Universal PCR primers and Index (X) Primer. PCR products were purified (AMPure XP system) and library quality was assessed on the Agilent Bioanalyzer 2100 system. Then, the clustering of the index-coded samples was performed on a cBot Cluster Generation System using TruSeq PE Cluster Kit v4-cBot-HS (Illumina, Inc., United States) according to the manufacturer’s instructions and the library preparations were sequenced on an Illumina Hiseq 2500 platform and single-end reads were generated.

For sRNA library construction, about 5 μg total RNA we used to construct sRNA library using the Small RNA Sample Prep Kit (Illumina). Since the sRNA has a phosphate and hydroxyl group at the 5′ and 3′ end, respectively, the T4 RNA ligase 1 and ligase 2 (truncated) were respectively ligated to corresponding ends of the sRNA. After ligation, the ligated RNA fragments were reverse transcribed using M-MLV reverse transcriptase (Invitrogen, Inc., United States) and then the resultant cDNA products were amplified with two primers corresponding to the ends of the adapter sequences. Polyacrylamide gel was used to get sRNA libraries by electrophoresis and rubber cutting recycling. Finally, to perform clustering and sequencing of sRNA, cluster generation was performed by using the TruSeq PE Cluster Kit v4-cBot-HS (Illumina, Inc., United States) according to the manufacturer’s protocols. After cluster generation, the library construction was loaded to an Illumina Hiseq 2500 platform and sequencing single-end reads were created.

Raw data with FASTQ format were analyzed through in-house Perl scripts. Clean reads were obtained by removing those reads containing adapter, ploy-N, and low-quality. Reads with smaller than 18 nt or longer than 30 nt were trimmed and cleaned. Meanwhile, several parameters of the clean data were calculated such as Q20, Q30, GC-content and sequence duplication level. Low-quality reads, such as only adaptor, unknown nucleotides >5%, or Q20 < 20% (percentage of sequences with sequencing error rates <1%), were removed by Perl script. All the downstream analyses were based on clean data with high quality. The clean reads filtered from the raw reads were mapped to *A. cerana* genome (Park et al., 2015; Diao et al., 2018b) and *A. mellifera* genome (Aml-4.5) (Elsik et al., 2014) using Tophat2 software. The aligned records from the aligners in BAM/SAM format were further examined to remove potential duplicate molecules. Gene abundance were calculated based on the value of the transcripts per million (TPM).

### Identification of Differential Gene Expression and Sequence Annotation

DESeq (Anders and Huber, 2010) and Q-value were employed to evaluate differential gene expression between CSBV and control groups. Gene expression levels were estimated using FPKM values (fragments per kilobase of exon per million fragments mapped) using Cufflinks software (Trapnell et al., 2010). The false discovery rate (FDR) control method was used to identify the threshold of the *P*-value in multiple tests to compute the significance of the differences. Here, only genes with an absolute value of log2 fold change ≥1.5 and FDR significance score <0.05 were used for subsequent analysis. Genes were compared against various protein databases by BLASTX, including the National Center for Biotechnology Information (NCBI) non-redundant protein (Nr) database, and Swiss-Prot database with a cut-off E-value of 10-5. Furthermore, genes were searched against the NCBI non-redundant nucleotide sequence (Nt) database using BLASTn by a cut-off E-value of 10-5. Genes were retrieved based on the best BLAST hit (highest score) along with their protein functional annotation. Differentially expressed honeybee genes were analyzed against a background set of genes, which are all the *Drosophila* orthologs (Drosophila genome vs. Dmel r5.42) in the honeybee genome (*A. cerana* and *A. mellifera* genome) (Elsik et al., 2014; Park et al., 2015; Diao et al., 2018b). The hierarchical clustering heatmap analysis of expression level of all genes was performed by MeV.^1^

### Identification of Small RNA Targeted Genes

Here, the sequences of ribosomal RNA (rRNA), transfer RNA (tRNA), small nuclear RNA (snRNA), small nucleolar RNA (snoRNA), and other kinds of non-coding RNAs were identified using a basic local alignment search tool (Bowtie tools soft, Version 1.1.2) against known non-coding RNAs deposited in the Silva database, GtRNAdb database, Rfam database and Repbase database, respectively, and unannotated reads of sRNA were obtained (Justin et al., 2014). MiRDeep2 software (Version 2.0.5) was mainly used for the prediction of animal miRNAs (Friedländer et al., 2012). Next, we mapped sRNA reads with lengths of 18–30 nt to the *A. cerana* genome (Park et al., 2015; Diao et al., 2018b) and CSBV genome (GenBank: KU574662.1) to get miRNA and CSBV-specific vsiRNA by using miRDeep2. Mapped reads were used to identify known miRNAs and novel miRNAs using miRBase 20.0 (Griffiths-Jones, 2006) and miREvo (Wen et al., 2012). Usually, these known miRNA data have been deposited in miRBase.^2^ Based on the distribution information of reads on the precursor sequence (miRNA production characteristics, mature, star, loop) and energy information of precursor structure (RNAfold randfold), the Bayesian model was used to score and authenticate the miRNAs and vsiRNA (Liu et al., 2009). We then classified these sRNAs into several different categories according to their annotations.

Last, we used psRobot^3^ to identify potential target genes of vsiRNAs in *A. cerana* (Wu et al., 2012), and used a well-established miRNA-target database MiRanda (v3.3a) and TargetScan (Version:7.0) to predict the target genes of the identified miRNAs, and investigated the possible functions using the method of gene function annotation (Agarwal et al., 2015). The vsiRNA profile was used as a query in sRNA target prediction module psRobot (Wu et al., 2012; see text footnote 3). Target gene function was annotated based on the following databases: Nr (NCBI non-redundant protein sequences); Nt (NCBI non-redundant nucleotide sequences); Pfam (Protein family); KOG/COG (Clusters of Orthologous Groups of proteins); Swiss-Prot (A manually annotated and reviewed protein sequence database); KO (KEGG Ortholog database); GO (Gene Ontology). The analysis of miRNA-mediated mRNA was performed based on the predicted miRNA-mRNA relationships. We selected miRNA-mRNA pairs showing opposite expression changes. The absolute value of log2 fold change ≥1 was used as a cutoff score.

### GO and KEGG Analysis

Genes were annotated in the NCBI non-redundant hits with GO (Conesa et al., 2005). Perl script was then used to plot GO functional classification for the genes with a GO term hit to view the distribution of gene functions. The obtained annotation was enriched and refined using TopGo (R package). The gene sequences were also aligned to the COG database to predict and classify functions (Tatusov et al., 2000). Kyoto Encyclopedia of Genes and Genomes (KEGG) pathways were assigned to the assembled sequences by Perl script. The interaction networks of corresponding proteins of targeted genes were predicted using STRING.^4^

### Verification of Expression Difference Genes by qPCR

For the analysis of miRNA expression, cDNA synthesis was performed using the miRCURY LNA Universal cDNA Synthesis kit II (Exiqon, Woburn, MA, United States) according to the manufacturer’s instructions and using specific reverse transcription primer (5′-CTCAACTGGTGTCGTGGAGTC GGCAATTCAGTTGAGCCCACCAA-3′) (Fu et al., 2012). Stem-loop reverse transcription quantitative PCR (qPCR) were performed as follows: 94° for 2 min followed by 30 cycles of 94°C for 30 s, 56°C for 35 s, and 72°C for 20 s. Specific sequence targeted vsiRNA 5′-ACACTCCAGCTGGGGCTGGGCCTTCTT ATTT-3′ was used as the forward primer, and URP, 5′-TGG TGTCGTGGAGTCG-3′ was used as the reverse primer. Data normalization of miRNA was carried out using the U6 reference gene (F: 5′-CTCGCTTCGGCAGCACA-3′, R: 5′-AACGCTTCACGAATTTGCGT-3′) (Yang et al., 2017).

Six immune genes were selected for RT-PCR validation to analyze the expression of mRNA related to immune of CSBV infection. The primers for the target genes are shown in Supplementary Table 2. Approximately 1,000 ng RNA was reverse transcribed using the PrimeScript RT Reagent Kit with gDNA Eraser (TaKaRa, Dalian, China) according to the manufacturer’s instructions, and the product was used as the template for qPCR. The qPCR reaction system consisted of 12.5 μL of 2× SYBR Premix Ex TaqTM II (Takara, Dalian, China), 0.5 μL of upstream and downstream primers (10 mM), respectively, 2 μL of template cDNA, and 9.5 μL of double-distilled H_2_O in a total volume of 25 μL. Real-time qPCR was performed using a cycling sequence of 95°C for 30 s, followed by 40 cycles of 95°C for 5 s, 54–56°C for 25 s, and 72°C for 25 s. β-actin was used as housekeeping gene and three technical replicates were performed. To verity the reliability of the RNA-seq result, twenty 2-day-old larvae were used to measure the expression of immune genes, while five larvae each of day-old (4th, 5th, 6^*th*^, and 7th) were used to quantify the expression of vsiRNA. Three replicates were run.

### Statistical Analysis

The limma algorithm was used to analyze the differentially expressed mRNAs and sRNAs according to the significant analysis with FDR analysis (Wettenhall et al., 2006). All data analysis meets the following two criteria: (1) log2 fold change ≥1.5 or *P* < 0.05; and (2) FDR < 0.05. The relative expression of the target genes in the infected and control groups were calculated with the 2^–ΔΔ*Ct*^ method. Statistical comparisons and Pearson correlation coefficient analysis were performed using GraphPad Prism 7 (GraphPad Software Inc., San Diego, CA, United States). Multiple *t*-tests were used to compare the significance of differential expression of genes between CSBV and control groups. The asterisks in the figures indicate significant differences (^∗^*P* < 0.05; ^∗∗^*P* < 0.01).

## Resuilts

### The Overview of Expressed mRNA and Small RNAs

A total of six RNA-seq libraries were obtained using Illumina sequencing platforms, but we subjected two pooled controls and two pooled CSBV infected samples to further analysis due to the two poor data with other virus contamination (data not show). For mRNA sample sequencing, a total of 18,270,587 (T01), 19,574,141 (T02), 17,646,189 (T03), and 20,618,929 (T04) clean reads were obtained from the healthy and CSBV-infected mRNA libraries (Supplementary Table 3) (T01 and T02, control groups; T03 and T04, e CSBV-infected group). Mapped reads to *A. cerana* genome were more than 70% among all samples (Supplementary Table 4). Meanwhile, for sRNA sequencing, a total of 18,731,778 (T01), 17,407,255 (T02), 15,937,441 (T03), and 13,237,265 (T04) clean reads were obtained from the healthy and CSBV-infected sRNA libraries (Supplementary Table 5). The value of Q30 was more than 90% among all samples, which indicated that these clean reads can be used for subsequent analysis (Supplementary Tables 3, 5).

**FIGURE 1:**
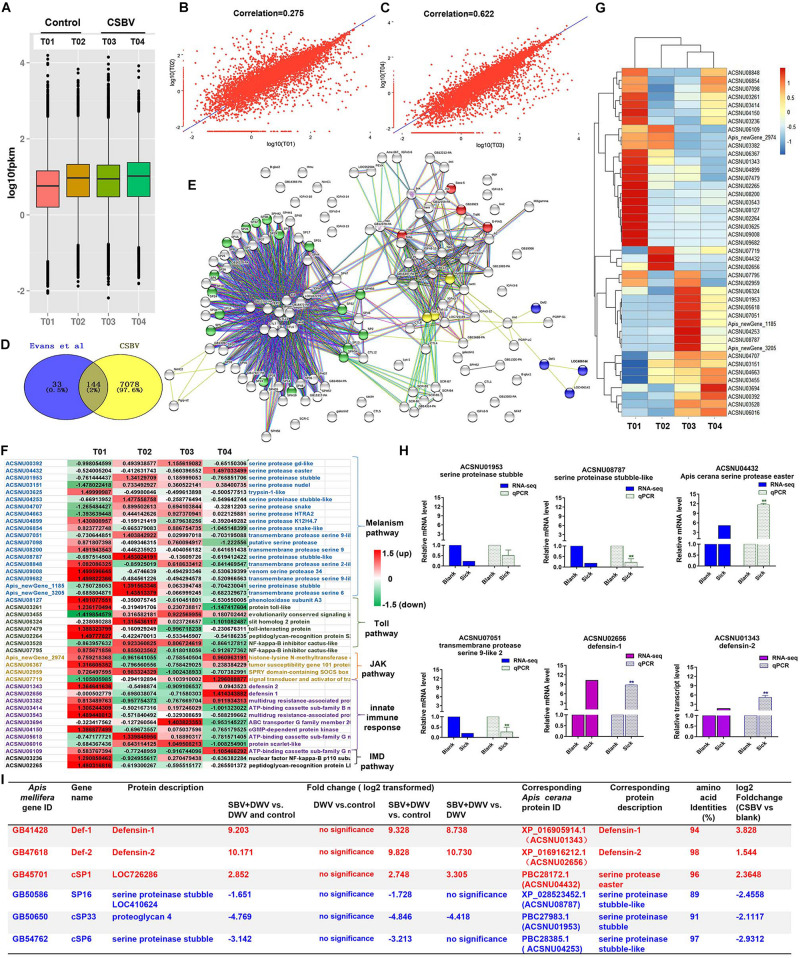
Summary of the types and features of genes from different immune pathways. **(A)** The analysis of expression level of samples based on FPKM value. T01 and T02, T03 and T04 indicate control and CSBV-infected groups. **(B)** Correlation analysis of the expression of all mRNA between healthy groups and CSBV-infected groups **(C)**. **(D)** The 144 differentially expressed immune genes were shared between our study and Evans et al. (2006) described. **(E)** The interaction network of 144 shared genes was predicted and found four major terms enriched (P < 0.05). Green, serine proteases and trypsin domain; red, Jak-STAT signaling pathway; blue, innate immune response; yellow, Toll receptor homology domain. **(F)** The analysis of the regulated genes related to serine proteases, melanization, and Toll pathway, Jak-STAT signaling pathway, innate immune response, and IMD pathway after CSBV infection. The values represent standardized expression levels based on FPKM mean values. Green and red indicate decreased and increased in expression levels, respectively. **(G)** Heat map of the regulated genes related to serine proteases, melanization, and Toll pathway, Jak-STAT signaling pathway, innate immune response, and IMD pathway. **(H)** The relative expression levels of serine proteinase and defensin genes from RNA-seq (T02, T03, and T04) and qPCR analyses. Actin was used as the internal reference gene. *P < 0.05, **P < 0.01. **(I)** The differentially expressed serine protease and defensin genes (*P < 0.05, FDR < 0.05) were compared with those of SBV-induced (Ryabov et al., 2016) (*P < 0.05, FDR < 0.05).

We further studied the expression levels of all mRNAs between control and CSBV-infected larvae based on FPKM value (Figure 1A), and then found that the FPKM value of mRNAs distributed from 0 to 10,000 in the four libraries, but the mean FPKM value in T01 was slightly lower than the other three libraries (Figure 1A). The correlation analysis on two pooled group using R.ggplot 2 confirmed that it is unreasonable to make T01 and T02 together in healthy group (*r* = 0.275) but reliable to make T03 and T04 together in CSBV-infected group (*r* = 0.622) (Figures 1B,C).

To detect the difference of immune response between healthy and CSBV-infected groups, comparative analysis was performed with the other studies. A comparison of our suite of 7,222 regulated genes (FDR < 0.05 and *P* < 0.05) and the 177 genes related to honeybee immune response and found that 144 genes were shared with those described by Evans and Armstrong(2006; Figure 1D). Then, we predicted the interaction networks of 144 genes and found 17 SPs genes (*P* < 0.0001) highly related to melanization pathway, four genes related to the JAK-STAT signaling pathway (*P* < 0.01), three Toll receptor homology domain genes (*P* < 0.05), and four genes involved in innate immune responses (*P* < 0.001), respectively (Figure 1E). The immune genes were up-regulated in T01 samples (Figure 1F), while heat map analysis confirmed that T01 was unsuitable to further analyze (Figure 1G). In addition, it is difficult to find healthy samples under natural conditions (Tan et al., 2007; Park and Yoe, 2017). Therefore, we used T02 as the control, T03 and T04 as CSBV-infected group to analyze the relative expression levels of defensin and serine proteinase genes (Figure 1H) and found that defensin 1 and 2 were significantly up-regulated (Figure 1H). The transcriptional levels of SP genes were variable. For example, serine proteinase stubble, serine proteinase stubble-like, and one transmembrane protease serine 9-like were down-regulated to 0. 5-, 0. 22-, and 0.26-fold, while SP easter had >11.68-fold elevated expression (Figure 1H), which was consistent with mRNA-seq analysis (*r* = 0.8004, *P* = 0.05) (Supplementary Figure 1).

Next, we performed comparative analysis of main immune responses of CSBV infections with the Ryabov et al. (2016) described. Our results showed that several differentially expressed genes including defensin and SP were down-regulated, which was consistent with those of Ryabov et al. (2016) reported (only P < 0.05, FDR < 0.05, and NCBI blast amino acid identities >80% were listed) (Figure 1I). For example, serine proteinase stubble-like and stubble genes were significantly down-regulated (P < 0.05) (Figure 1H). Additionally, qPCR results showed that the expression of the prophenoloxidase (ppo) gene was significantly down-regulated at the 4-, 5-, 6-, and 7-day-old after CSBV infection (Supplementary Figure 2).

### GO and KEGG Analysis on Differentially Expressed mRNA Involved in Response to CSBV Infection

**FIGURE 2:**
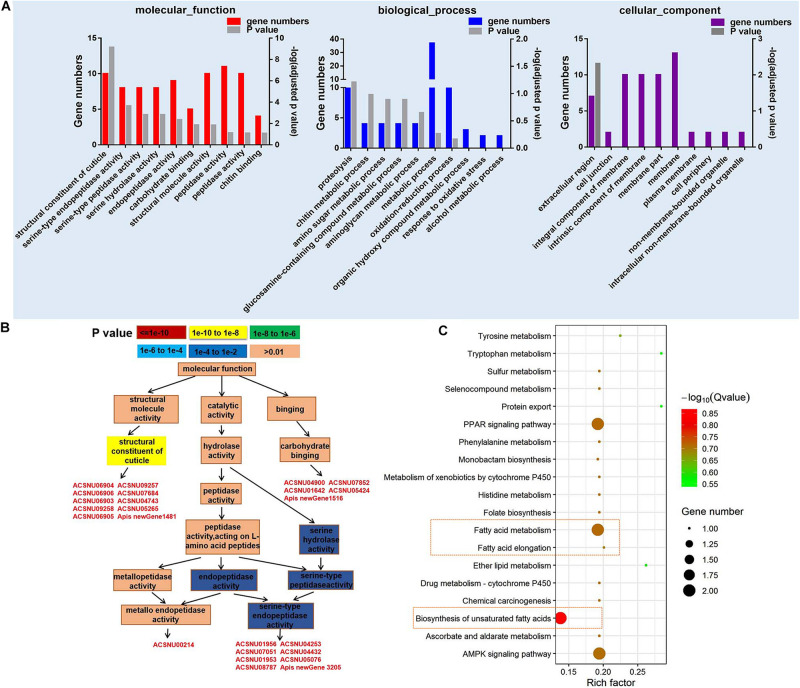
GO and KEGG pathway enrichment analysis of differentially expressed genes (DEGs). **(A)** The top 10 GO terms of DEGs in molecular function, biological process, and cellular component (*P* < 0.05). **(B)** GO-directed acyclic graph indicates the term of molecular function. The color represents the significance levels in enrichment according to *P*-value. **(C)** The KEGG pathway enrichment analysis of DEGs (*P* < 0.05).

A total of 10,262 mRNA transcripts were produced (FDR < 0.05) (Supplementary Table 6), and 203 significantly differently expressed genes (DEGs) (Supplementary Table 7). The cutoff of log2-fold change >1.5 and *P*-value > 0.8 was used to filter genes that were differentially expressed between CSBV-infected and the control samples (Tarazona et al., 2011). Among the 203 DEGs, 83 genes were successfully matched to GO terms (Figure 2A), and the top 10 GO terms (*P* ≤ 0.01) of molecular function belong to the structural constituent of cuticle, serine-type endopeptidase, serine-type peptidase, and serine hydrolase activity (Figure 2A). Based on GO-directed acyclic graph the relationship among these 10 terms in molecular function showed that the structural constituent of cuticle and serine-type endopeptidase activity were significantly enriched (*P* < 0.01) (Figure 2B). Ten genes related to the structural constituent of cuticle were significantly up-regulated including larval cuticle protein A1A, larval cuticle protein A2B, flexible cuticle protein 12-like, and larval cuticle protein A3A (Supplementary Table 7). While seven SP genes were significantly down-regulated except *easter* was up-regulated.

Kyoto Encyclopedia of Genes and Genomes analysis on 203 DEGs found that fatty acid metabolism, biosynthesis of unsaturated fatty acids, AMP-activated protein kinase (AMPK) signaling pathway, and peroxisome proliferator-activated receptors (PPAR) signaling pathway were mainly enriched in 20 categories (Figure 2C). Two acyl-CoA Delta 11-desaturase-like genes (ACSNU05780 and ACSNU05780) involving in fatty acid metabolism and PPAR signaling pathway were significantly up-regulated to fourfold. Furthermore, we also found that two genes related to fatty acid biosynthesis were significantly down-regulated to 0.12 fold at least (Supplementary Table 7).

### GO Analysis and KEGG Annotation on Target mRNA of Differentially Expressed miRNA

**FIGURE 3:**
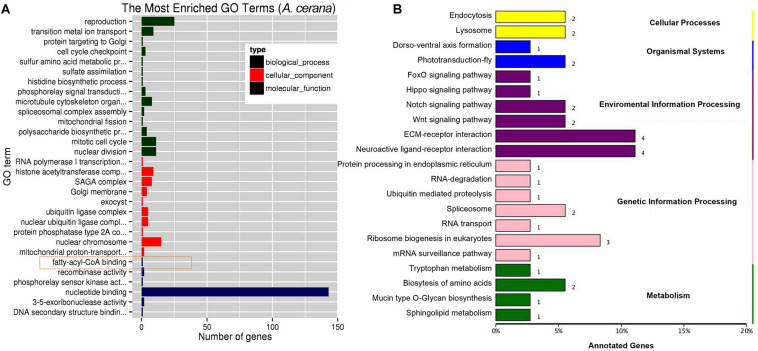
GO and KEGG annotation on target genes of differentially expressed miRNA. **(A)** The top GO terms enriched (*P* < 0.05) on target genes of differentially expressed miRNA in molecular function, biological process and cellular component. **(B)** The KEGG analysis of target genes of differentially expressed miRNA (*P* < 0.05).

Using miRDeep2 and DESeq software, we mapped clean sRNA reads to the *A. cerana* genome against the miRanda database (Park et al., 2015; Diao et al., 2018b). The compositions of those reads of sRNA are shown in Supplementary Figure 3, and the most abundant class of sRNAs were 22-nt in size, including known miRNA and new identified miRNA (Supplementary Figures 3A,B). The 260 miRNAs were obtained included 23 differently expressed miRNAs (Supplementary Table 8, log2 fold change >0.6 or <−0.6, *P* < 0.05). The 260 miRNAs were targeted 1035 mRNAs and 23 differently expressed miRNAs were predicated toward 227 target mRNAs that involved in biological process, cellular component, and molecular function (Figure 3A). KEGG annotation analysis showed that 227 target genes mainly participate in ECM-receptor interaction, ribosome biogenesis in eukaryotes and endocytosis (Figure 3B).

We identified a miRNA, ame-miR-3759, was significantly up-regulated to more than onefold (Table 1), while the expression of the two target genes, LOC410515 and putative uncharacterized protein DDB_G0277255-like, were significantly down-regulated (*P* < 0.01). LOC410515 may have a similar function in immune with a homologous protein, *Apis dorsata* chorion peroxidase-like (XP_006611124.1) (NCBI blast sequence identify >75%), which played an important role in melanin synthesis (Feng et al., 2018).

**TABLE 1:** The opposite expression in miRNA-mRNA pairs.

miRNA	miRNA_log2 fold change	Target mRNA	mRNA_log2 fold change	Annotation	Homologous protein
ame-miR-3759	1.09	ACSNU05809	−3.40	DDB_G0277255-like	Peptidyl-prolyl cis-trans isomerase
ame-miR-3759	1.09	ACSNU05034	−3.61	LOC410515	Chorion peroxidase

### Identification and Analysis on Target mRNA of CSBV-Specific siRNA

**FIGURE 4:**
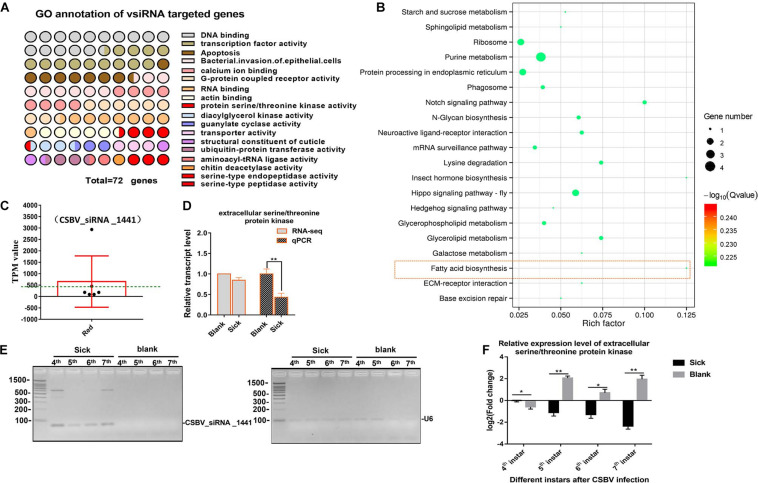
GO and KEGG annotation of vsiRNAs target genes. **(A)** GO annotation of vsiRNAs target genes in CSBV infected larvae. **(B)** The KEGG analysis of vsiRNAs target genes. **(C)** The TPM value of vsiRNA_1441 targeted extracellular serine/threonine protein kinase FAM20C-like gene of Apis (the TPM value was higher than mean value, green dash line). **(D)** The comparison on relative expression level of the serine/threonine protein kinase FAM20C obtained from RNA-seq and qPCR verified. **(E)** Identification on constant expression of vsiRNA_144 at different day-old of CSBV-infected larvae. U6 RNA is used as a loading control. **(F)** The comparison on relative expression levels of serine/threonine protein kinase FAM20C between CSBV-infected and control larvae at different day-old. *P < 0.05,**P < 0.01.

We aligned all the cleaned sRNA-seq reads to the CSBV genome (GenBank: KU574662.1) and we identified 319 out of 2,467 unique vsiRNAs targeted 290 genes. Most of targets genes were enriched in involved in DNA binding, transcription factor activity, apoptosis, G-protein coupled receptor activity, transporter activity, and SP activity (Figure 4A and Supplementary Table 10). KEGG analysis on target mRNA of CSBV-specific siRNA showed that purine metabolism, hippo signaling pathway and fatty acid biosynthesis were significantly enriched (*P* < 0.05) (Figure 4B).

Although the expression of Dicer-like and Argonaute-2 (Ago2) genes associated to the RNAi pathway were up-regulated (Supplementary Table 5 and Supplementary Figure 4), we focused on siRNAs related to serine/threonine kinase, serine-type endopeptidase and serine-type peptidase genes based on the analysis of mRNA and miRNA data. Generally, the complete expression profile of vsiRNAs was categorized into “low” (TPM value <50), “high” (TPM value ≥50), and “very high expression” (TPM value ≥1,000) (relative abundance) based on the TPM value of siRNAs (Ramesh et al., 2017). For example, the expression levels of vsiRNA_1441 (TPM value is 2,932) were extremely significant higher than other siRNAs (3–20 times higher, mean value is 405) (Figure 4C and Supplementary Table 11). Then, we found that the target mRNA of vsiRNA_1441 was extracellular serine/threonine protein kinase FAM20C-like of Apis, which potentially regulate many processes including cell survival, growth and metabolism. Subsequently, qPCR analysis confirmed that the expression of extracellular serine/threonine protein kinase FAM20C was significantly down-regulated (Figure 4D). Next, the expressions of vsiRNA_1441 of CSBV-infected larvae at different day-old were identified by RT-PCR (Figure 4E). Similarly, the expression of the extracellular serine/threonine protein kinase FAM20C gene was significantly down-regulated at the 4-, 5-, 6-, and 7-day-old after CSBV infection (Figure 4F).

## Discussion

Healthy larvae are crucial for the development and growth of the *A. cerana* colony population. However, CSBV was considered as one of factors contributed to recent declines of the *A. cerana* colony (Diao et al., 2018b). A few studies have shown that vsiRNAs negatively regulate host mRNAs and effectively silence host genes to gain more proliferation (Yang et al., 2018). Here, to understand the immune response of the CSBV infection, we presented the first sRNA analysis to CSBV under natural condition. The RNA-seq results showed that 203 DEGs were significantly altered between the healthy and infected larvae. Of these, all cuticle protein genes related to structural constituent of cuticle were significantly up-regulated, while SP genes related to serine-type endopeptidase activity, serine-type peptidase activity and serine hydrolase activity were significantly down-regulated. Meanwhile, 23 differently expressed miRNAs and 319 effective vsiRNAs were identified, which some of vsiRNAs targeted serine/threonine kinase or serine-type endopeptidase-related genes. These findings provide a novel insight into how CSBV resists host immune responses and result in larvae unable to the pupae.

Cuticle proteins might play a key role in resistance to CSBV infection. Cuticle is a barrier against viral invasion in animals such as white spot syndrome virus infection in shrimp (Corteel et al., 2009). Cuticle proteins play a critical role in keeping the body from pathogens and serving as a barrier to resistance them (Zhao et al., 2017). It has been reported that natural resistance-associated macrophage protein (NRAMP) was a cellular receptor of Sindbis virus in insects (Rose et al., 2011). In aphidiae, cuticle protein 4 is essential to entry of cucumber mosaic virus (CMV) because it was considered as a viral putative receptor (Liang and Gao, 2017). Although NRAMP was not enriched in our study, our results showed that the expression levels of larvae cuticle protein genes were greatly elevated including larval cuticle protein A2B, larval cuticle protein A1A and larval cuticle protein A3A. While Ryabov et al. (2016) found that the genes involved in cuticle and muscle development were down-regulated at a later stage after SBV infection (9 dpi). This was similar with that deformed wing virus infection will induce a cuticular protein down-regulated, apidermin 3 (Tomas et al., 2019). Thus, we infer that cuticle protein may have an important role to resistance CSBV infection during the development of larvae.

Serine proteases were significantly inhibited by CSBV infection. SPs with 60–400 members in insects form a large family of enzymes that hydrolyze peptide bonds at different rates (Rawlings and Barrett, 1993; Cao et al., 2017). SPs and SPHs, especially extracellular SPs, have a great influence on insect development and innate immunity (Ahola et al., 2015; Dudzic et al., 2019). Experimental evidence showed that SPs of *A. mellifera* may be involved in the embryonic development, melanization, and immune responses (Zou et al., 2006; Rodriguez-Andres et al., 2012). Our results suggested that most immunity genes related to the SPs were overlapped with Evans’ study (Evans and Armstrong, 2006) and two SPs, serine-type endopeptidase and peptidase activity, were significantly down-regulated. RT-qPCR results confirmed also that CSBV infection caused down-regulation of SP genes, such as SP stubble and transmembrane protease serine 9. This was consistent with that of sacbrood virus infection (Ryabov et al., 2016).

Honeybees can initiate humoral immunity involving SPs that include coagulation, melanization, and the Toll signal pathway (Rodriguez-Andres et al., 2012). SPs exist in hemolymph in the form of zymogens and then activate downstream genes such as *ppo*. Although Gao et al. (2017) identified an SP gene, *Accsp10*, related to development and pathogens resistance of *A. cerana*, they did not confirm its function. However, our results showed that the *ppo* gene of melanization pathway were down-regulated. Additionally, we also found that the expression level of the *ppo* gene was significantly down-regulated at the 4-, 6-, and 7-day-old in CSBV-infected larvae and suggested that CSBV infection significantly suppressed the melanization response by inhibiting the expression of SP and PPO. This was similar to that of semliki forest virus inhibited on the phenoloxidase cascade of mosquito (Rodriguez-Andres et al., 2012). However, intermediate steps of CSBV infection inhibited the SPs need to be further investigated.

Small RNAs have been confirmed to be related to a diversity of biological processes including cell proliferation and apoptosis through the post-transcriptional regulation of gene expression (Charkhpour et al., 2011; Zhang et al., 2014; Yang et al., 2017). RNA profile analyses suggested that these sRNAs were involved in biological processes associated with development and immunity of honeybees (Chen et al., 2017). The key initiator of the RNAi pathway is dsRNA, which produced by proliferation process are processed into vsiRNA duplexes by dicer-2 (Xu and Zhou, 2017). Viral small interfering RNAs are processed into 21-nt through Dicer-2 on viral dsRNA and then are integrated into insect Ago2 and guide the Ago2 onto target RNAs to cause their degradation (Bartholomay and Michel, 2018). It has also been reported that vsiRNAs can regulate the gene expression related to the host RNAi pathway and enhance the pathogenesis. For example, Xu et al. (2017) found that siRNAs derived from Southern rice black-streaked dwarf virus targeted several types of genes related to host resistance such as receptor-like protein kinases. Chejanovsky et al. (2014) identified the viral siRNA population from honeybee colony with colony collapse disorder and indicated those siRNA responses were against viral infection. Recently, Chen et al. (2014) feed siRNA target the viral suppressor of RNAi and significantly suppressed IAPV replication. In our study, a total of 290 vsiRNAs targeted mRNA were related to DNA binding, transcription factor activity, apoptosis, G-protein coupled receptor activity, transporter activity, serine/threonine kinase activity and serine-type endopeptidase, and suggested various vsiRNA could affect the expression of host genes by RNAi pathway. For instance, the expression of Dicer-like and Ago2 genes were up-regulated. Especially, vsiRNA_1441 significantly suppressed the expression of extracellular serine/threonine protein kinase FAM20C-like.

Previous studies investigated mainly host response to viral infection. However, metabolites are another important indicator that can reflect physiological response to pathogens (Marelli-Berg et al., 2012). Fatty acid metabolism and biosynthesis are known to play a vital role in viral infections and proliferation (Fritsch and Weichhart, 2016; Wang et al., 2019). It has reported that fatty acids can influence host immune by affecting the inflammatory repertoire of the host, substrates for biosynthesis of inflammatory mediators and activation of cell receptors (Huang et al., 2012). In our study, KEGG analysis showed that 203 DEGs were significantly enriched in fatty acid metabolism and biosynthesis, which was found in accordance with integrated KEGG analysis of DEGs and vsiRNA target mRNA. In addition, two acyl-CoA Delta 11-desaturase-like genes related to fatty acid metabolism and biosynthesis of unsaturated fatty acids were significantly up-regulated after CSBV infection, while two long chain fatty acids genes were significantly down-regulated. It was reported that fatty acids were used to control the American foulbrood and other bee diseases of honeybee (Kuzyšinová et al., 2016). These evidences suggested that fatty acid metabolism and biosynthesis in host immune response could be critical factors to resistance CSBV. However, further studies are needed to clarify the detail process.

According to result from this study combined with previous studies, we propose that CSBV deploys sRNAs to modulate honeybee immune by targeting genes associated with multiple biological processes. One, down-regulated serine/threonine protein kinase and serine-type endopeptidase are key genes of SP cascade and affect melanization response. The other is to down-regulate the expression of acyl-CoA Delta 11-desaturase and long chain fatty acids genes, progress to disruption of the fatty acid biosynthesis and metabolism. Our study offers important insights into understanding the mechanism of pathogenicity of CSBV and may lead to new molecular tools for both viral diagnosis and control. However, further studies will test the functions of SPs and cuticle proteins during the viral infection.

## Data Availability Statement

The datasets generated or analyzed during the current study are available from the public database, http://gsa.big.ac.cn/(CRA002310).

## Author Contributions

CH conceived and revised the manuscript. YD, HZ, SS, SY, DY, and SD performed the experiments. YD analyzed the data and wrote the manuscript. All authors contributed to the article and approved the submitted version.

## Conflict of Interest

The authors declare that the research was conducted in the absence of any commercial or financial relationships that could be construed as a potential conflict of interest.

## Footnotes

1 https://sourceforge.net/projects/mev-tm4/

2 http://www.mirbase.org

3 http://omicslab.genetics.ac.cn/psRobot/

4 http://string.embl.de/

## Supplementary Material

Supplementary Figure 1 The correlation analysis (*r* value = 0.8, *P-*value = 0.05) between qPCR validation and RNA-seq data to identify the reliability of the result.

Supplementary Figure 2 Expression level of *ppo* gene at different day-old after CSBV infection. ^∗∗^*P* < 0.01.

Supplementary Figure 3 The length distribution of sRNA. **(A)** The length distribution of known miRNA **(B)**, new identified miRNA **(C)** and CSBV-specific siRNA (vsiRNA).

Supplementary Figure 4 Expression level of genes related to RNAi pathway based on FPKM value.

Supplementary Table 1 Primers used for detection the presence of common honey bee viruses.

Supplementary Table 2 Primers used to run qPCR for target genes of siRNA.

Supplementary Table 3 The list of genes and their annotations from identified mRNA between control and CSBV infected larvae.

Supplementary Table 4 The comparison on sequence data between our sequencing data and reference genome.

Supplementary Table 5 The analysis of sRNA sequencing data.

Supplementary Table 6 Summary for the transcriptome responses between control and CSBV-infected larvae.

Supplementary Table 7 The list of target genes and their annotations from 203 differential expressed mRNA between normal and CSBV infected larvae.

Supplementary Table 8 23 differential expressed miRNAs between control and CSBV infected larvae.

Supplementary Table 9 The list of target genes of siRNAs from CSBV-infected larvae.

Supplementary Table 10 The annotations on target genes of siRNAs from CSBV-infected larvae.

Supplementary Table 11 The TPM value of identified siRNAs from CSBV-infected larvae.

## References

[B1] Agarwal V. Bell G. W. Nam J. W. Bartel D. P. (2015). Predicting effective microRNA target sites in mammalian mRNAs. *eLife* 4:e05005. 10.7554/eLife.05005 26267216 PMC4532895

[B2] Ahola V. Koskinen P. Wong S. C. Kvist J. Paulin L. Auvinen P. (2015). Temperature- and sex-related effects of serine protease alleles on larval development in the Glanville fritillary butterfly. *J. Evol. Biol.* 28 2224–2235. 10.1111/jeb.12745 26337146

[B3] Ai H. Yan X. Han R. (2012). Occurrence and prevalence of seven bee viruses in *Apis mellifera* and *Apis cerana* apiaries in china. *J. Invertebr. Pathol.* 109 160–164. 10.1016/j.jip.2011.10.006 22062807

[B4] Aliyari R. Wu Q. Li H. W. Wang X.-H. Li F. Green L. D. (2008). Mechanism of induction and suppression of antiviral immunity directed by virus-derived small RNAs in *Drosophila*. *Cell Host Microbe* 4 387–397. 10.1016/j.chom.2008.09.001 18854242 PMC2584229

[B5] Anders S. Huber W. (2010). Differential expression analysis for sequence count data. *Genome Biol.* 11:R106. 10.1186/gb-2010-11-10-r106 20979621 PMC3218662

[B6] Azzami K. Ritter W. Jürgen T. Beier H. (2012). Infection of honey bees with acute bee paralysis virus does not trigger humoral or cellular immune responses. *Arch. Virol.* 157 689–702. 22258854 10.1007/s00705-012-1223-0 PMC3314816

[B7] Bailey L. Gibbs A. J. Woods R. D. (1964). Sacbrood virus of the larval honey bee (*Apis mellifera* linnaeus). *Virology* 5 425–429. 10.1016/0042-6822(64)90266-1 14194138

[B8] Bartholomay L. C. Michel K. (2018). Mosquito immunobiology: the intersection of vector health and vector competence. *Annu. Rev. Entomol.* 63 145–167. 29324042 10.1146/annurev-ento-010715-023530

[B9] Boncristiani H. F. Evans J. D. Chen Y. Pettis J. Murphy C. Lopez D. L. (2013). *In vitro* infection of pupae with Israeli acute paralysis virus suggests disturbance of transcriptional homeostasis in honey bees (*Apis mellifera*). *PLoS One* 8:e73429. 10.1371/journal.pone.0073429 24039938 PMC3764161

[B10] Brutscher L. M. Daughenbaugh K. F. Michelle L. F. (2015). Antiviral defense mechanisms in honey bees. *Curr. Opin. Insect Sci.* 10 71–82. 10.1016/j.cois.2015.04.016 26273564 PMC4530548

[B11] Campbell E. M. Budge G. E. Watkins M. Bowman A. S. (2015). Transcriptome analysis of the synganglion from the honey bee mite, *Varroa destructor* and RNAi knockdown of neural peptide targets. *Insect Biochem. Mol. Biol.* 70 116–126. 10.1016/j.ibmb.2015.12.007 26721201

[B12] Cao X. Gulati M. Jiang H. (2017). Serine protease-related proteins in the malaria mosquito, *Anopheles gambiae*. *Insect Biochem. Mol. Biol.* 88 48–62. 10.1016/j.ibmb.2017.07.008 28780069 PMC5586530

[B13] Charkhpour M. Samadi H. Bahari B. Parvizpur A. (2011). Evaluation of the effect of intracerebroventricular injection of a1-adenosine receptors agonist on withdrawal syndrome of morphine in rats. *Eur. Neuropsychopharmacol.* 21(Suppl. S3), S584–S585.

[B14] Chejanovsky N. Ophir R. Schwager M. S. Slabezki Y. Grossman S. Cox-Foster D. (2014). Characterization of viral siRNA populations in honey bee colony collapse disorder. *Virology* 454–455 176–183. 10.1016/j.virol.2014.02.012 24725944

[B15] Chen X. Ma C. Chen C. Lu Q. Shi W. Liu Z. (2017). Integration of lncRNA-miRNA-mRNA reveals novel insights into oviposition regulation in honey bees. *PeerJ* 5:e3881. 10.7717/peerj.3881 29018616 PMC5632538

[B16] Chen Y. P. Pettis J. S. Corona M. Chen W. P. Li C. J. Spivak M. (2014). Israeli acute paralysis virus: epidemiology, pathogenesis and implications for honey bee health. *PLoS Pathog.* 10:e1004261. 10.1371/journal.ppat.1004261 25079600 PMC4117608

[B17] Chen Y. P. Siede R. (2007). Honey bee viruses. *Adv. Virus Res.* 70 33–80. 10.1016/S0065-3527(07)70002-7 17765703

[B18] Conesa A. Gotz S. Garcia-Gomez J. M. Terol J. Talon M. Robles M. (2005). Blast2GO: a universal tool for annotation, visualization and analysis in functional genomics research. *Bioinformatics* 21, 3674–3676. 10.1093/bioinformatics/bti610 16081474

[B19] Cornman R. S. Bennett A. K. Murray K. D. Evans J. D. Elsik C. G. Aronstein K. (2012). Transcriptome analysis of the honey bee fungal pathogen, *Ascosphaera apis*: implications for host pathogenesis. *BMC Genomics* 13:285. 10.1186/1471-2164-13-285 22747707 PMC3425160

[B20] Corteel M. Dantas-Lima J. J. Wille M. Alday-Sanz V. Pensaert M. B. Sorgeloos P. (2009). Molt stage and cuticle damage influence white spot syndrome virus immersion infection in penaeid shrimp. *Vet. Microbiol.* 137 209–216. 10.1016/j.vetmic.2009.01.018 19201551

[B21] Deng Y. C. Zhao H. X. Shen S. Yang S. Yang D. H. Deng S. (2020). mRNA and small RNA-seq reveal insights into immune regulation in *Apis cerana* after Chinese Sacbrood virus infection. *BioRxiv* [Preprint]. 10.1101/2020.07.07.192799

[B22] Diao Q. Li B. Zhao H. Wu Y. Guo R. Dai P. (2018a). Enhancement of chronic bee paralysis virus levels in honeybees acute exposed to imidacloprid: a Chinese case study. *Sci. Total Environ.* 630 487–494. 10.1016/j.scitotenv.2018.02.258 29499530

[B23] Diao Q. Sun L. Zheng H. Zeng Z. Wu J. (2018b). Genomic and transcriptomic analysis of the Asian honeybee *Apis cerana* provides novel insights into honeybee biology. *Sci. Rep.* 8:822. 10.1038/s41598-017-17338-6 PMC5770391 29339745

[B24] Ding S. W. (2010). RNA-based antiviral immunity. *Nat. Rev. Immunol.* 10 632–644. 10.1038/nri2824 20706278

[B25] Ding S. W. Voinnet O. (2007). Antiviral immunity directed by small RNAs. *Cell* 130 413–426. 10.1016/j.cell.2007.07.039 17693253 PMC2703654

[B26] Dudzic J. P. Hanson M. A. Iatsenko I. Kondo S. Lemaitre B. (2019). More than black or white: melanization and toll share regulatory serine proteases in drosophila. *Cell Rep.* 27 1050–1061. 10.1016/j.celrep.2019.03.101 31018123

[B27] Elsik C. G. Worley K. C. Bennett A. K. Beye M. Camara F. Childers C. P. (2014). Finding the missing honey bee genes: lessons learned from a genome upgrade. *BMC Genomics* 15:86. 10.1186/1471-2164-15-86 24479613 PMC4028053

[B28] Evans J. D. Armstrong T. N. (2006). Antagonistic interactions between honey bee bacterial symbionts and implications for disease. *BMC Ecol.* 6:4. 10.1186/1472-6785-6-4 16551367 PMC1471774

[B29] Evans J. D. Aronstein K. Chen Y. Hetru C. Imler J. Jiang H. (2006). Immune pathways and defense mechanisms in honey bees *Apis mellifera*. *Insect Mol. Biol.* 15 645–656. 10.1111/j.1365-2583.2006.00682.x 17069638 PMC1847501

[B30] Feng D. Li Q. Yu H. Kong L. Du S. (2018). Transcriptional profiling of long non-coding RNAs in mantle of *Crassostrea gigas* and their association with shell pigmentation. *Sci. Rep.* 8:1436. 10.1038/s41598-018-19950-6 PMC5780484 29362405

[B31] Flenniken M. L. Andino R. (2013). Non-specific dsRNA-mediated antiviral response in the honey bee. *PLoS One* 8:e77263. 10.1371/journal.pone.0077263 24130869 PMC3795074

[B32] Fletcher S. J. Anita S. Peters J. R. Carroll B. J. Rajagopalbabu S. Pappu H. R. (2016). The tomato *Spotted wilt virus* genome is processed differentially in its plant host *Arachis hypogaea* and its thrips vector *Frankliniella fusca*. *Front. Plant Sci.* 7:1349. 10.3389/fpls.2016.01349 27656190 PMC5013717

[B33] Francis R. M. Nielsen S. L. Kryger P. (2012). Patterns of viral infection in honey bee queens. *J. Gen. Virol.* 94(Pt 3), 668–676. 23223622 10.1099/vir.0.047019-0 PMC3709610

[B34] Friedländer M. R. Mackowiak S. D. Li N. Chen W. Rajewsky N. (2012). miRDeep2 accurately identifies known and hundreds of novel microRNA genes in seven animal clades. *Nucleic Acids Res.* 40 37–52. 10.1093/nar/gkr688 21911355 PMC3245920

[B35] Fritsch S. D. Weichhart T. (2016). Effects of interferons and viruses on metabolism. *Front. Immunol.* 7:630. 10.3389/fimmu.2016.00630 28066439 PMC5174094

[B36] Fu Y. S. Shi Z. Y. Wang G. Y. Li W. J. Zhang J. L. Jia L. (2012). Expression and regulation of mir-1, -133a, -206a, and MRFs by thyroid hormone during larval development in paralichthys olivaceus. *Comp. Biochem. Phys. B.* 161, 226–232. 10.1016/j.cbpb.2011.11.009 22142802

[B37] Fung E. Hill K. Hogendoorn K. Glatz R. Napier K. Bellgard M. (2018). *De novo* assembly of honey bee RNA viral genomes by tapping into the innate insect antiviral response pathway. *J. Invertebr. Pathol.* 152 38–47. 10.1016/j.jip.2018.01.002 29378202

[B38] Galbraith D. A. Yang X. Niño E. L. Yi S. Grozinger C. (2015). Parallel epigenomic and transcriptomic responses to viral infection in honey bees (*Apis mellifera*). *PLoS Pathog.* 11:e1004713. 10.1371/journal.ppat.1004713 25811620 PMC4374888

[B39] Gao L. Wang H. Liu Z. Liu S. Zhao G. Xu B. (2017). The initial analysis of a serine proteinase gene (*AccSp10*) from *Apis cerana cerana*: possible involvement in pupal development, innate immunity and abiotic stress responses. *Cell Stress Chaperones* 22 867–877. 28695333 10.1007/s12192-017-0818-5 PMC5655375

[B40] Ghosh R. C. Ball B. V. Willcocks M. M. Carter M. J. (1999). The nucleotide sequence of sacbrood virus of the honey bee: an insect picorna-like virus. *J. Gen. Virol.* 80(Pt 6), 1541–1549. 10374974 10.1099/0022-1317-80-6-1541

[B41] Griffiths-Jones S. (2006). Mirbase: the microRNA sequence database. *Methods Mol. Biol.* 342 129–138. 16957372 10.1385/1-59745-123-1:129

[B42] Han B. Zhang L. Feng M. Fang Y. Li J. (2013). An integrated proteomics reveals pathological mechanism of honeybee (*Apis cerana*) sacbrood disease. *J. Proteome Res.* 12 1881–1897. 10.1021/pr301226d 23418745

[B43] Hu Y. Fei D. Jiang L. Wei D. Li F. Diao Q. (2016). A comparison of biological characteristics of three strains of Chinese sacbrood virus in *Apis cerana*. *Sci. Rep.* 6:37424. 10.1038/srep37424 27853294 PMC5112594

[B44] Huang S. Rutkowsky J. M. Snodgrass R. G. Ono-Moore K. D. Schneider D. A. Newman J. W. (2012). Saturated fatty acids activate TLR-mediated proinflammatory signaling pathways. *J. Lipid Res.* 53 2002–2013. 10.1194/jlr.D029546 22766885 PMC3413240

[B45] Justin C. Sara S. Anthony R. Jackman S. D. Ming N. K. Richard M. (2014). Biobloom tools: fast, accurate and memory-efficient host species sequence screening using bloom filters. *Bioinformatics* 30 3402–3404. 10.1093/bioinformatics/btu558 25143290 PMC4816029

[B46] Kuzyšinová K. Mudroòová D. Toporèák J. Molnár L. Javorsky P. (2016). The use of probiotics, essential oils and fatty acids in the control of American foulbrood and other bee diseases. *J. Apicult. Res.* 55 386–395. 10.1080/00218839.2016.1252067

[B47] Li W. Evans J. D. Huang Q. Rodríguez-García C. Liu J. Hamilton M. (2016). Silencing the honey bee (*Apis mellifera*) naked cuticle gene (n.d.) improves host immune function and reduces *Nosema ceranae* infections. *Appl. Environ. Microbiol.* 82 6779–6787. 27613683 10.1128/AEM.02105-16 PMC5086571

[B48] Liang Y. Gao X. W. (2017). The cuticle protein gene mpcp4 of *Myzus persicae* (Homoptera: Aphididae) plays a critical role in cucumber mosaic virus acquisition. *J. Econ. Entomol.* 110 848–853. 10.1093/jee/tox025 28334092

[B49] Liu H. Wang Z. Tian L. Q. Qin Q. H. Wu X. B. Yan W. Y. (2014). Transcriptome differences in the hypopharyngeal gland between Western honeybees (*Apis mellifera*) and Eastern honeybees (*Apis cerana*). *BMC Genomics* 15:744. 10.1186/1471-2164-15-744 25174638 PMC4158095

[B50] Liu H. Yue D. Zhang L. Chen Y. Gao S. J. Huang Y. (2009). “A computational framework to study public health epidemiology,” in *Proceedings of the International Joint Conference on Bioinformatics, Systems Biology and Intelligent Computing*, Shanghai, 10.1109/IJCBS.2009.83

[B51] Liu S. Wang L. H. Guo J. Tang Y. J. Chen Y. P. Wu J. (2017). Chinese sacbrood virus infection in Asian honey bees (*Apis cerana cerana*) and host immune responses to the virus infection. *J. Invertebr. Pathol.* 150 63–69. 10.1016/j.jip.2017.09.006 28916146

[B52] Ma M. Liu J. Song Y. Li L. Li Y. (2013). TaqMan MGB probe fluorescence real-time quantitative PCR for rapid detection of Chinese Sacbrood virus. *PLoS One* 8:e52670. 10.1371/journal.pone.0052670 23408931 PMC3568131

[B53] Ma M. Liu M. Jian C. Song Y. Shude W. L. P. (2011). Molecular and biological characterization of Chinese sacbrood virus LN isolate. *Comp. Funct. Genomics* 2011:409386. 10.1155/2011/409386 21527980 PMC3061217

[B54] Marelli-Berg F. M. Fu H. Mauro C. (2012). Molecular mechanisms of metabolic reprogramming in proliferating cells: implications for T-cell-mediated immunity. *Immunology* 136 363–369. 10.1111/j.1365-2567.2012.03583.x 22384794 PMC3401974

[B55] Park D. Jung J. W. Choi B. S. Jayakodi M. Lee J. Lim J. (2015). Uncovering the novel characteristics of Asian honey bee, *Apis cerana*, by whole genome sequencing. *BMC Genomics* 16:1. 10.1186/1471-2164-16-1 25553907 PMC4326529

[B56] Park S. I. Yoe S. M. (2017). Defensin-like peptide3 from black solder fly: identification, characterization, and key amino acids for anti-gram-negative bacteria. *Entomol. Res.* 47 41–47. 10.1111/1748-5967.12214

[B57] Ramesh S. V. Williams S. Kappagantu M. Mitter N. Pappu H. R. (2017). Transcriptome-wide identification of host genes targeted by tomato *Spotted wilt virus*-derived small interfering RNAs. *Virus Res.* 238 13–23. 10.1016/j.virusres.2017.05.014 28545854

[B58] Rawlings N. D. Barrett A. J. (1993). Evolutionary families of peptidases. *Biochem. J.* 290(Pt 1), 205–218. 10.1042/bj2900205 8439290 PMC1132403

[B59] Rodriguez-Andres J. Rani S. Varjak M. Chase-Topping M. E. Beck M. H. Ferguson M. C. (2012). Phenoloxidase activity acts as a mosquito innate immune response against infection with Semliki forest virus. *PLoS Pathog.* 8:e1002977. 10.1371/journal.ppat.1002977 23144608 PMC3493465

[B60] Rose P. Hanna S. Spiridigliozzi A. Wannissorn N. Beiting D. Ross S. (2011). Natural resistance-associated macrophage protein is a cellular receptor for *Sindbis virus* in both insect and mammalian hosts. *Cell Host Microbe* 10 97–104. 10.1016/j.chom.2011.06.009 21843867 PMC3164510

[B61] Rutter L. Carrillo-Tripp J. Bonning B. C. Cook D. Toth A. L. Dolezal A. G. (2019). Transcriptomic responses to diet quality and viral infection in *Apis mellifera*. *BMC Genomics* 20:412. 10.1186/s12864-019-5767-1 31117959 PMC6532243

[B62] Ryabov E. V. Fannon J. M. Moore J. D. Wood G. R. Evans D. J. (2016). The iflaviruses sacbrood virus and deformed wing virus evoke different transcriptional responses in the honeybee which may facilitate their horizontal or vertical transmission. *PeerJ* 4:e1591. 10.7717/peerj.1591 26819848 PMC4727977

[B63] Sheyn U. Rosenwasser S. Lehahn Y. Barakgavish N. Rotkopf R. Bidle K. D. (2018). Expression profiling of host and virus during a coccolithophore bloom provides insights into the role of viral infection in promoting carbon export. *ISME J.* 8 704–713. 10.1038/s41396-017-0004-x 29335637 PMC5864229

[B64] Siede R. Meixner M. D. Büchler R. (2012). Comparison of transcriptional changes of immune genes to experimental challenge in the honey bee (*Apis mellifera*). *J. Apicult. Res.* 51 320–328. 10.3896/IBRA.1.51.4.05

[B65] Sun L. Li M. Fei D. Diao Q. Wang J. Li L. (2018). Preparation and application of egg yolk antibodies against Chinese sacbrood virus infection. *Front. Microbiol.* 9:1814. 10.3389/fmicb.2018.01814 30123212 PMC6085425

[B66] Tan S. Gan G. Paeper B. Proll S. Katze M. G. (2007). Systems biology and the host response to viral infection. *Nat. Biotechnol.* 25 1383–1389. 10.1038/nbt1207-1383 18066032 PMC7097743

[B67] Tarazona S. Garcia-Alcalde F. Dopazo J. Ferrer A. Conesa A. (2011). Differential expression in RNA-seq: a matter of depth. *Genome Res.* 21 2213–2223. 10.1101/gr.124321.111 21903743 PMC3227109

[B68] Tatusov R. L. Galperin M. Y. Natale D. A. Koonin E. V. (2000). The COG database: a tool for genome-scale analysis of protein functions and evolution. *Nucleic Acids Res.* 28 33–36. 10.1093/nar/28.1.33 10592175 PMC102395

[B69] Tomas E. Bruno S. Klara K. Pavel T. Karel H. (2019). *Varroa destructor* parasitism has a greater effect on proteome changes than the deformed wing virus and activates TGF-β signaling pathways. *Sci. Rep.* 9: 9400. 10.1038/s41598-019-45764-1 PMC6599063 31253851

[B70] Trapnell C. Williams B. A. Pertea G. Mortazavi A. Kwan G. van Baren M. J. (2010). Transcript assembly and quantification by RNA-Seq reveals unannotated transcripts and isoform switching during cell differentiation. *Nat. Biotechnol.* 28 511–515. 10.1038/nbt.1621 20436464 PMC3146043

[B71] Wang J. Liu J. Y. Shao K. Y. Han Y. Q. Li G. Ming S. L. (2019). Porcine reproductive and respiratory syndrome virus activates lipophagy to facilitate viral replication through downregulation of NDRG1 expression. *J. Virol.* 93:e00526-19. 10.1128/JVI.00526-19 PMC6694807 31189711

[B72] Wen M. Shen Y. Shi S. Tang T. (2012). miREvo: an integrative microRNA evolutionary analysis platform for next-generation sequencing experiments. *BMC Bioinformatics.* 13:140. 10.1186/1471-2105-13-140 22720726 PMC3410788

[B73] Wettenhall J. M. Simpson K. M. Satterley K. Smyth G. K. (2006). Affylmgui: a graphical user interface for linear modeling of single channel microarray data. *Bioinformatics* 22 897–899. 10.1093/bioinformatics/btl025 16455752

[B74] Wu H. J. Ma Y. K. Chen T. Wang M. Wang X. J. (2012). PsRobot: a web-based plant small RNA meta-analysis toolbox. *Nucleic Acids Res.* 40 W22–W28. 22693224 10.1093/nar/gks554 PMC3394341

[B75] Xu D. Zhou G. (2017). Characteristics of siRNAs derived from *Southern rice black-streaked dwarf virus* in infected rice and their potential role in host gene regulation. *Virol. J.* 14:27. 10.1186/s12985-017-0699-3 PMC5301327 28183327

[B76] Yang M. Xu Z. Zhao W. Liu Q. Li Q. Lu L. (2018). Rice stripe virus-derived siRNAs play different regulatory roles in rice and in the insect vector *Laodelphax striatellus*. *BMC Plant Biol* 18:219. 10.1186/s12870-018-1438-7 30286719 PMC6172784

[B77] Yang R. Dong B. Fan R. Guo Z. Li X. Hong T. (1988). Investigation of nucleic acids and polypeptides of Chinese sacbrood virus. *Virol. Sin.* 1 54–58.

[B78] Yang Y. Xie Y. Wu M. Geng Y. Li R. Xu L. (2017). Expression of mmu-miR-96 in the endometrium during early pregnancy and its regulatory effects on stromal cell apoptosis via Bcl2. *Mol. Med. Rep.* 15 1547–1554. 10.3892/mmr.2017.6212 28259902 PMC5364990

[B79] Zhang W. Yan L. Li Y. Chen W. Hu N. Wang H. (2014). Roles of miRNA-24 in regulating endothelial nitric oxide synthase expression and vascular endothelial cell proliferation. *Mol. Cell. Biochem.* 405 281–289. 10.1007/s11010-015-2418-y 25920448

[B80] Zhao X. Gou X. Qin Z. Li D. Wang Y. Ma E. (2017). Identification and expression of cuticular protein genes based on *Locusta migratoria* transcriptome. *Sci. Rep.* 7:45462. 10.1038/srep45462 28368027 PMC5377371

[B81] Zou Z. Lopez D. L. Kanost M. R. Evans J. D. Jiang H. (2006). Comparative analysis of serine protease-related genes in the honey bee genome: possible involvement in embryonic development and innate immunity. *Insect Mol. Biol.* 15 603–614. 10.1111/j.1365-2583.2006.00684.x 17069636 PMC1761132

