# Supplementary figures and images for: Identification of Immune Response to Sacbrood Virus Infection in Apis cerana Under Natural Condition
Source: Front Genet. 2020 Oct 26;11:587509. doi: 10.3389/fgene.2020.587509 (PMC7649357; doi:10.3389/fgene.2020.587509)

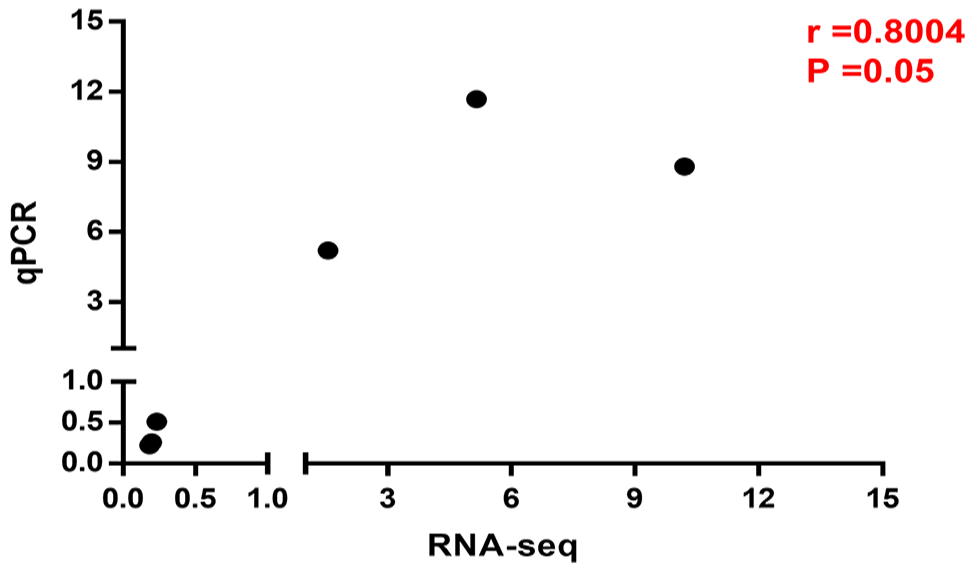

Supplement: Supplementary Figure 1 — The correlation analysis (r value = 0.8, P-value = 0.05) between qPCR validation and RNA-seq data to identify the reliability of the result. [file Image_1.TIF]

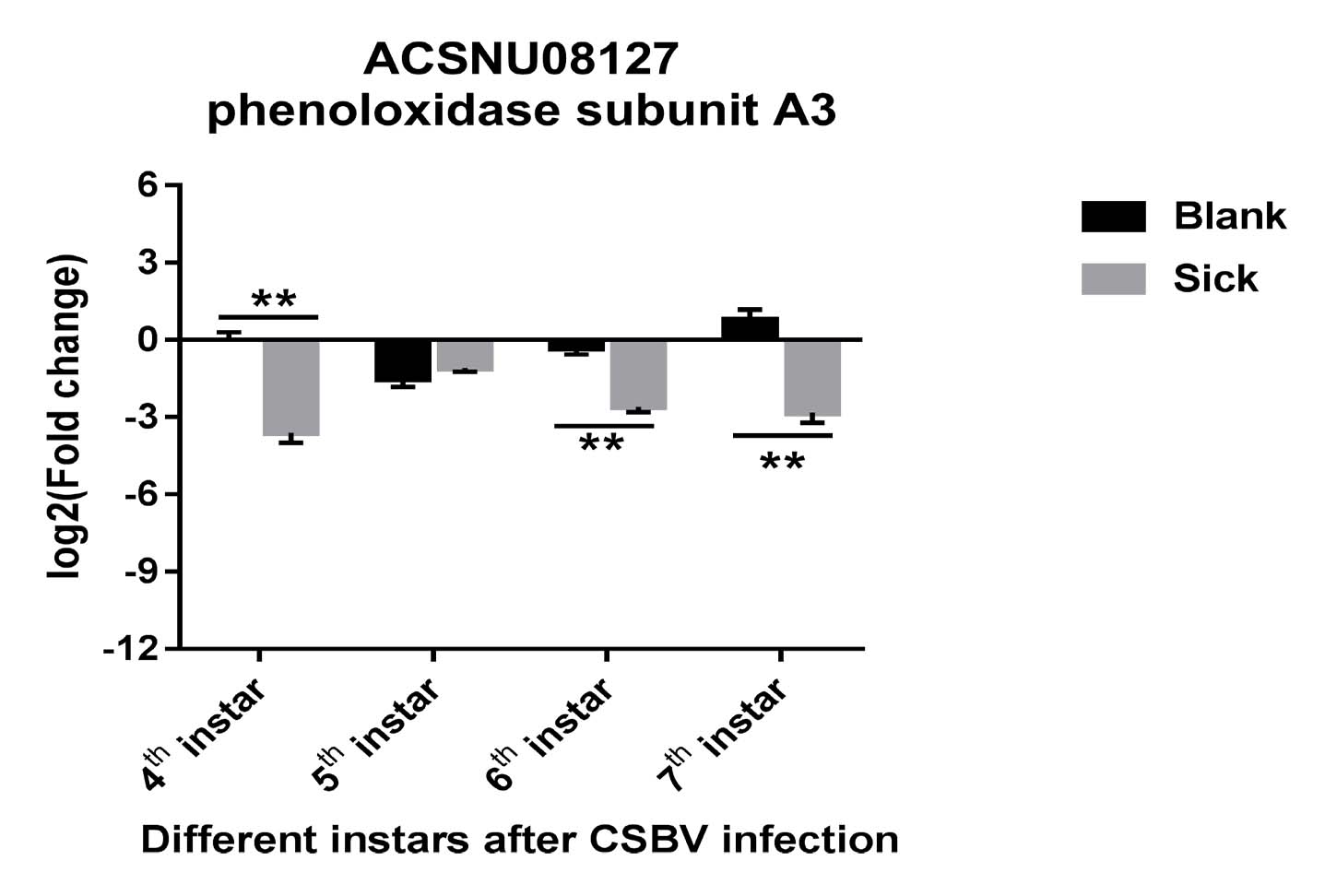

Supplement: Supplementary Figure 2 — Expression level of ppo gene at different day-old after CSBV infection. ∗∗P < 0.01. [file Image_2.TIF]

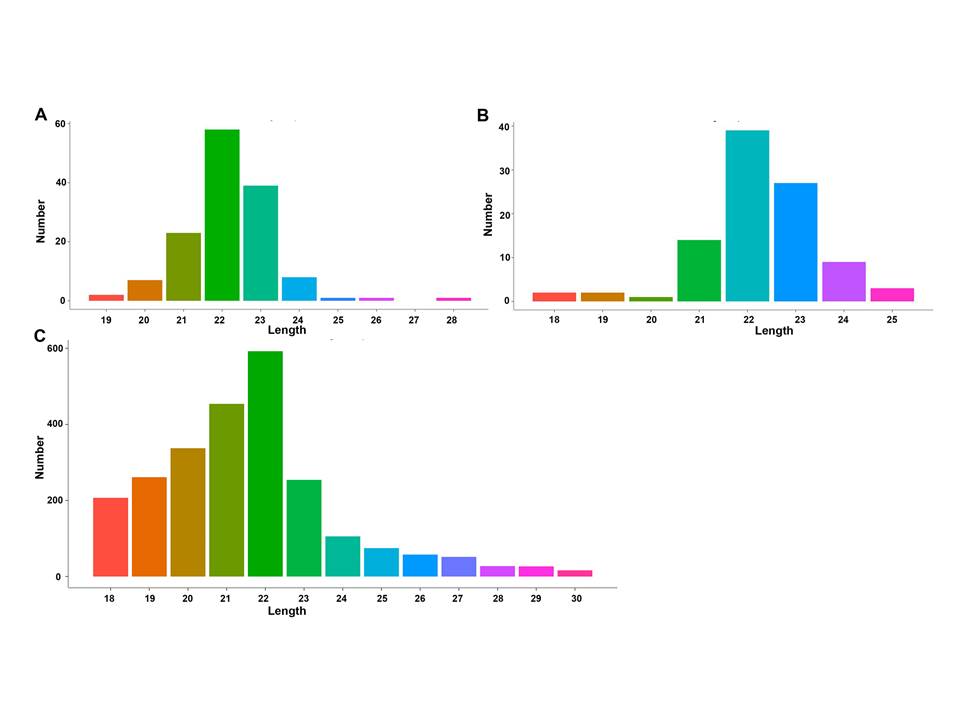

Supplement: Supplementary Figure 3 — The length distribution of sRNA. (A) The length distribution of known miRNA (B), new identified miRNA (C) and CSBV-specific siRNA (vsiRNA). [file Image_3.TIF]

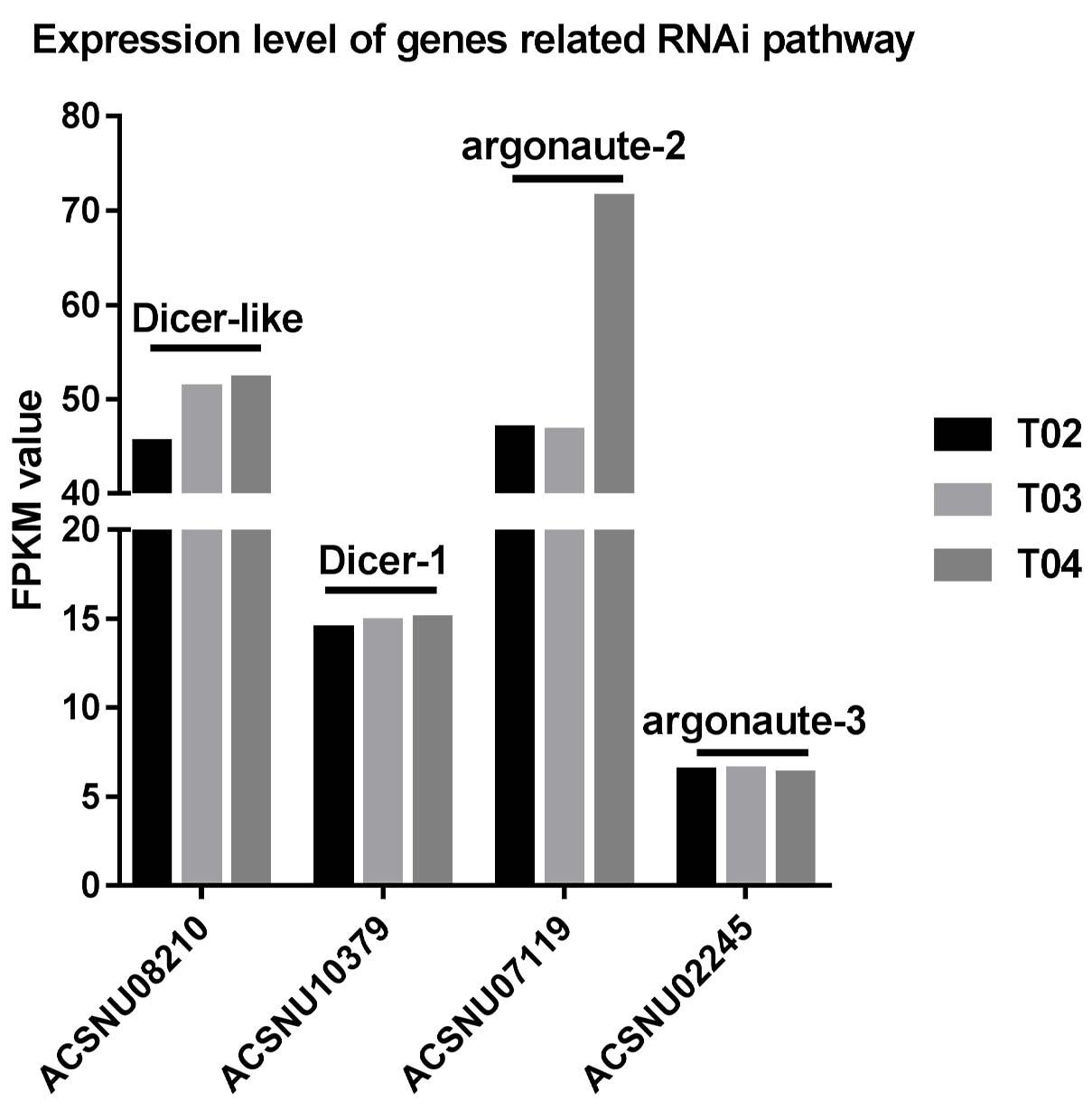

Supplement: Supplementary Figure 4 — Expression level of genes related to RNAi pathway based on FPKM value. [file Image_4.TIF]
